# Haptic Edge Detection Through Shear

**DOI:** 10.1038/srep23551

**Published:** 2016-03-24

**Authors:** Jonathan Platkiewicz, Hod Lipson, Vincent Hayward

**Affiliations:** 1 The City College of New York, The City University of New York, Department of Mathematics, New York, NY 10031, USA; 2 Columbia University, Department of Mechanical Engineering, New York, NY 10027, USA; 3 Sorbonne Universités, UPMC Univ Paris 06, UMR 7222, ISIR, F-75005, Paris, France

## Abstract

Most tactile sensors are based on the assumption that touch depends on measuring pressure. However, the pressure distribution at the surface of a tactile sensor cannot be acquired directly and must be inferred from the deformation field induced by the touched object in the sensor medium. Currently, there is no consensus as to which components of strain are most informative for tactile sensing. Here, we propose that shape-related tactile information is more suitably recovered from shear strain than normal strain. Based on a contact mechanics analysis, we demonstrate that the elastic behavior of a haptic probe provides a robust edge detection mechanism when shear strain is sensed. We used a jamming-based robot gripper as a tactile sensor to empirically validate that shear strain processing gives accurate edge information that is invariant to changes in pressure, as predicted by the contact mechanics study. This result has implications for the design of effective tactile sensors as well as for the understanding of the early somatosensory processing in mammals.

A reliable mapping between the physical world and the acquired data is a basic issue faced by any artificial or natural sensory system. For instance, in vision, a fundamental challenge is to access robustly the geometry of a body from the structure of the captured light intensity, despite variations in the viewing conditions. This task is difficult because the intrinsic geometry of a body is not mapped one-to-one to the geometry of images^1^,^2^,^3^. Solutions to this problem have many applications, chief among them is computer vision^4^. Insights into this process also have important consequences for our understanding of the neurophysiology of visual perception^5^,^6^. In haptics, an analogous problem arises when attempting to access shape information from the deformation of a sensing probe in contact with an unknown rigid object^7^. In vision, analysis of the variations in distribution of light intensity enables the projected image to be decomposed into a set of primitives—edges, bars, blobs, and terminations—giving rise to the so-called ‘raw primal sketch’^8^. This decomposition leads to the notion of scale-space, where an image is analyzed at different resolutions since similar structures may occur at different scales^9^,^10^,^11^,^12^, thus enabling scale-invariant feature detection and analysis^13^. Given appropriate constraints, the primal sketch makes the identification of key aspects of the three-dimensional structure of a scene not only possible, but robust and efficient.

Here, it is proposed that a similar concept is applicable to touch, subject to the differences in the type of available sensory data in the two modalities. The regions of high strain caused by the protruding, low curvature features of a touched solid are undoubtedly of key relevance to haptic perception at different stages of somatosensory processing^14^,^15^. The robust detection of regions of high strain is thus of fundamental importance in the elaboration of any intermediate shape representation. In this study, we highlight an intriguing similarity between the inverse problems that must be solved in haptic shape processing and in visual shape processing when considering the mechanical behavior of an elastic medium deformed by an unknown object.

Artificial tactile sensors are typically comprised of an elastic sheet set against a rigid foundation in which mechanically sensitive elements are embedded^16^,^17^,^18^,^19^,^20^,^21^,^22^,^23^. Several mechanical parameters have been proposed for being tactually relevant: surface deformation, normal strain or stress, shear strain or stress, principal strains or axial strains, or a combination of these parameters. Currently, there is no consensus as to which of these parameters the sensors should detect (see a recent review^24^).

In primate fingers, which are very soft, the assumption that a high firing rate of the afferents innervating a given region of skin is a sufficient representation of an edge is rarely questioned^25^. However, the match with *in vivo* data has generally required the fit of models associated to numerous assumptions and parameters; and has been achieved for only certain contact conditions^26^,^27^,^28^. These discrepancies, often attributed to the complexities of skin mechanics, may also be a reflection of the fact that at the length-scale of the mechanoreceptors, the macroscopic laws of continuum mechanics do not apply, and the receptors exhibit sensitivity to privileged modes of deformation^7^.

In the present study, we propose that shear strain is a highly informative quantity to be extracted from a sensing sheet because the information that it contains is largely invariant to contact conditions. This hypothesis is justified by a mechanical analysis of the contact between an object and an elastic medium. The theory was tested using the universal jamming gripper^29^,^30^. Taking advantage of its shape-memory property, this device was employed as a tactile sensor by imaging its surface after contact with an object.

## Results

### Why shear?

**Figure 1:**
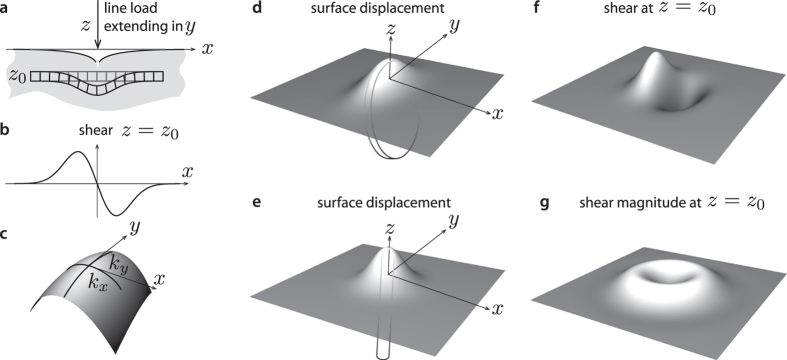
Shear sensing hypothesis. (**a**) First consider an elastic half-space indented by a line load (knife-edge). The black line shows surface displacement according to the classical theory^38^. Surface displacement is undefined at *x* = 0 in the case of an infinitely narrow edge. A deformation field develops inside the elastic body. The gray and black blocks respectively represent undeformed and deformed cubic elements. Shear strain corresponds to the angular deformation of these elements. (**b**) The graph shows the evolution of shear along the *x* direction. (**c**) Generic contact patch with the axes aligned with the principal curvatures, *k*_*x*_ and *k*_*y*_, the inverses of the radii of the osculating circles. Surface displacement caused (**d**) by an edge of finite extent, such as the edge of a coin where *k*_*x*_ is large and *k*_*y*_ small, and (**e**) by a small axi-symmetric punch where *k*_*x*_ and *k*_*y*_ are both high. (**f**) Regions of shear develop on each side of the impinging edge. (**g**) For a small punch, the region of shear deformation forms an annulus centered where it is zero. If the curvature of the indenting object becomes small, the magnitude plot of the shear morphs into the case of the punch.

The shear sensing hypothesis has been advanced by several authors^31^,^32^,^33^. Using the finite-element method, Ricker and Ellis pointed out that shear strain enables one to distinguish contacts causing similar normal strain profiles. Using a contact mechanics analysis, Wang and Hayward suggested that the shear strain distribution resulting from a line load resembles the derivative of a Gaussian function. In effect, line loads induce shear strain distributions that exhibit local maxima on each side of a narrow contact while going through zero where the edge impinges on the surface (Fig. 1a,b), irrespective of the magnitude of the pressure.

When a rigid object with protruding edges comes in contact with an elastic half-space, the contacts can be modeled in first approximation as a line load or punch indentation according to the magnitude of the principal curvatures in the local region of contact. For instance, in human fingers, ‘quasi infinite edges’ arise if one curvature of the object is greater than that of the finger and if the other curvature is much smaller. Curved or short edges with high and low principal curvatures, Fig. 1c, like those of a small coin (or of the tip of a screwdriver), will create deformation fields that resemble those shown in Fig. 1d. These edges create contact conditions where the magnitude of the shear has local maxima in two regions, indicating the presence of an edge where shear crosses zero, see Fig. 1f. Another frequent case is that of the punch indentation, such as a protruding corner or a Braille dot (high local principal curvatures), see Fig. 1e. These cases correspond to the formation of an annulus of shear around the point of contact. Shear strain crosses zero along all radial directions, see Fig. 1g.

These local extrema and zero crossings can serve as descriptive primitives of the underlying signal in a very general way^34^. In haptic processing we propose that the local extrema of the distribution of shear can be robust descriptors of the local topography of a touched object. In a given domain, pairs of extrema of shear strain would reveal the presence of an edge and its orientation under the condition that local shear crosses zero. A detection method based on such generic features would be invariant to surface pressure, making the detection robust to contact conditions.

### Edges in vision and touch

In vision, edges correspond to discontinuities in the light intensity. These discontinuities can arise from the occlusion of an object by another, discontinuities in surface orientation or reflectance, or cast shadows. These circumstances create edges in a great variety of cases. In touch, edges primarily indicate discontinuities in surface orientation, and only if these discontinuities are protruding. They are rarely caused by other contact instances. Notably, sharp concave edges cannot be touched and must be inferred^35^. As a result, the principal curvatures must always be of the same sign. Flush surfaces with discontinuous frictional properties, such as different materials assembled edge-to-edge, are not typically classified as tactile edges (see later a discussion about their illusory occurrence), while their putative visual equivalents—illumination discontinuities on a uniform surface—are certainly classified as genuine edges.

**Figure 2:**
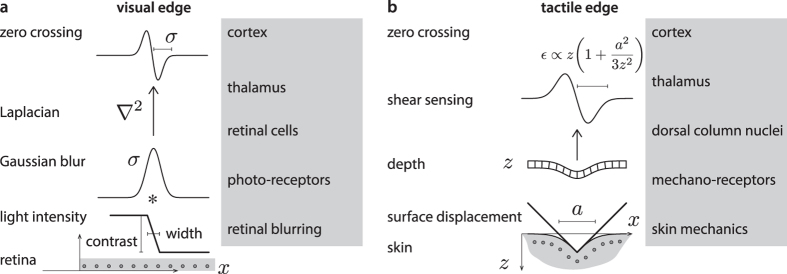
Visual edges compared to tactile edges. The detection of a step cannot be performed by direct differentiation because it is an ill-posed problem^56^. Sensor signals being invariably corrupted by noise, including sampling noise, differentiation is inherently unstable. (**a**) In machines and animals it is well accepted that edge detection must begin by a smoothing operation (convolution, ‘*’, by a Gaussian) to set the scale, *σ*, at which the signal ought to be processed according to contrast and width. The ability to handle multiple scales is a fundamental requirement to achieve invariance with respect to distance and illumination. This step is followed by double differentiation to locate the edge by detection of local extrema and zero crossings. In animals, this process begins in the retinal cells and is distributed in the visual pathway and the cortical primary visual areas. (**b**) In touch, since individual sensing units are set at a distance, *z*, from the surface, the detection of a roof begins by blurring owing to the elastic mechanics of the sensor. If, according to our hypothesis, shear deformation is sensed then edges can be located robustly, like in vision, by the detection of local extrema and zero crossings, without the need to differentiate twice. The mechanical processing scale is determined by the depth of the receptors and the depth of indentation. Scale invariance should apply to indentation depth since the same edge must be detected regardless of how much pressure is applied. In mammals, this process could begin in tactile afferents^54^, continue in the dorsal column nuclei^55^, and terminate in the cortical somatosensory areas^60^.

Beyond the greater variety of situations corresponding to visual edges compared to tactile edges, the notion of edge differs more profoundly in the two modalities. Discontinuities in a light field are primarily described by step discontinuities, that is, when the light intensity differs in magnitude on each side of an edge. While roof discontinuities can occasionally arise in vision, in touch, edges are always roof discontinuities in sensor surface displacement (Fig. 2b). Even in the limit case of a knife-edge or sharp punch indentation, the surface displacement must remain continuous, lest the sensor be damaged; it is the surface orientation that is discontinuous and differs on each side of an edge. Because mechanical sensing cannot be performed at the probe’s surface, contact mechanics dictates that what can be sensed must be a blurred transformation of surface displacement (an exception must be made for optically-based tactile sensors that convert mechanical roof discontinuities into optical step discontinuities^36^).

In vision, it is well accepted that edges can be extracted from an image by filtering it with a Laplacian of Gaussian function (Fig. 2a)^10^,^37^. We propose that tactile edges can be extracted by a similar process, but the implementation of this process must differ fundamentally in the two modalities. Mechanically, it is possible to express shear strain, *γ*, in terms of the Laplacian of Gaussian filters (*Methods*). Thus, whenever a narrow contact impinges on the surface of a tactile probe, the condition, *γ* = 0, must be satisfied just below the surface. This result can be seen by locating a narrow contact at the origin, *x* = 0, and expressing the shear field, *γ*(*x*, *z*), as the convolution of the surface pressure, *p*(*x*), by a Gaussian of width *ε*, *ϕ*_*ε*_(*x*), differentiated along the spatial variable, *x*, scaled by depth, *z*, and by the inverse of the elastic modulus of the medium, *E*, (*Methods*),


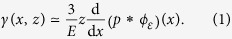


It implies that in terms of displacements, if *w*(*x*) is the surface displacement normal to the contact plane, the condition,


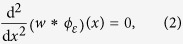


must hold for an incompressible elastic medium. The function, *ϕ*_*ε*_(*x*), acts like ‘spread function’ from the view point of sensing units embedded in the surrounding elastic medium. From the limit case of a knife edge, and the Taylor expansion of the solution of the Boussinesq-Flamant’s problem^38^ (*Methods*), we can deduce that the blurring width is 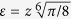 , which thus scales with depth in the medium. Finite angle roof edges, blunt edges, and round edges will generally give rise to the same blurring property as sharp edges provided that the local details of high curvature are smoothed by the mechanics. For example, in the case of roof edges of support 2*a* in contact with the surface, Fig. 2b, the equivalent blur width is 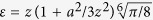. Thus, the dullness of the edge operates as a correction term (*Methods*). An edge with an obtuse angle will appear more blurred than a sharp edge. In all cases, the deeper the mechanoreceptors are located, the blurrier is their vision of the surface.

Expression (1) considers the ideal case of a frictionless surface, but realistic interactions generally also include a tangential component, the traction, *q*(*x*). Considering the contributions of both pressure and traction gives (*Methods*),


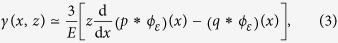


which shows that certain types of interactions other than edges can create shear zero-crossings, further suggesting that tactile sensing, as noted earlier, is inherently ambiguous.

**Figure 3:**
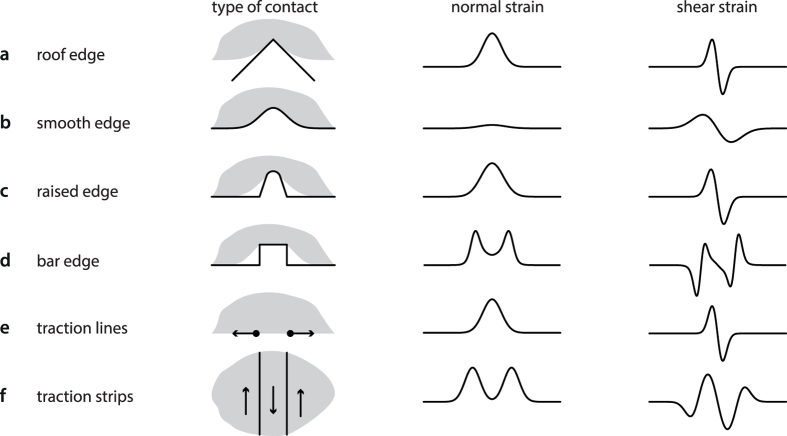
Comparison of the information provided by normal vs. shear strain sensing. (**a**) The common roof edge does correspond to a extremum in normal strain. The detection of this extremum, however, is mathematically equivalent to the computation of the point where its derivative is zero, the computation of which is an ill-posed problem^56^. The value of its maximum increases with indentation and thus lacks an important invariant property. In contrast, shear strain crosses zero for any indentation depth at the edge location. (**b**) A smooth edge corresponds to a weak, undetectable variation of normal strain. The zero-crossing of shear strain is conserved regardless of the local geometry of the edge. (**c**) A raised edge has a sufficiently small curvature such that, once blurred, it is detected like a sharp edge despite lack of conformability. (**d**) A bar, or double edge, corresponds to two maxima in an appropriate range of scales. Shear strain preserves the zero crossings for a wide range of bar geometries and sensor depths. Cases (**a**,**b**,**d**) were considered in^32^,^40^,^61^. The first term of (3) suggests that if pressure is an even function then shear strain is an odd function as in cases (**a**–**d**). For pure differential traction, case (**e**) could be confused with (**a**) owing to the second term yielding the same profile of shear as the first term in case (**a**) for appropriate values of the spacing and up to a 90° rotation^33^. The two cases are felt similarly^47^. (**f**) Strips imposing alternating tractions produce below the surface normal strains and shear strains that vary similarly since the first term is zero in this case^50^. The central strip is felt as protruding (or recessing) from the surface as in case (**d**)^49^,^50^.

Despite these ambiguities, in typical cases, shear analysis enables the robust detection of the presence and the localization of an edge, whereas normal strain measurement leads to solving an ill-posed problem (Fig. 3a–d). The complexity of the mechanical equations enables the existence of ambiguous contact conditions that cannot be resolved by normal strain or shear strain sensing, see Fig. 3e,f.

### Predictions relative to haptic sensing

The application of contact mechanics theory to the interaction of a soft probe with a rigid object, (3), allows us to draw important conclusions regarding the haptic sensing of shape:

- The elasticity of the probe creates a Gaussian filter of scale, 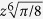, with this quantity receiving a correction term dependent on the edge sharpness. This implies that the deeper the sensors, the more blurred the effects of surface deflection; the zero-crossings are preserved at all depths just below the surface.
- Stiffer medium decreases tactile sensitivity; resolution is independent of the stiffness of the medium. Protecting the sensing units from damage limits how close they can be to the surface, which can be compensated by a softer material.
- In the absence of friction, the elastic behavior of a probe can give rise to a Laplacian of Gaussian filter of the surface displacement normal to the contact plane. It follows that, according to the fingerprint theorem^39^, this displacement can be recovered if the shear strain is known at different depths below the surface of the probe.
- Tangential displacements and normal indentation can cause similar sensory inputs. For example, if 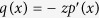 (*Methods*), certain pressure distributions and tangential tractions distributions give rise to the same shear magnitudes. The same is true of normal strain.

### Experimental detection of an edge

**Figure 4:**
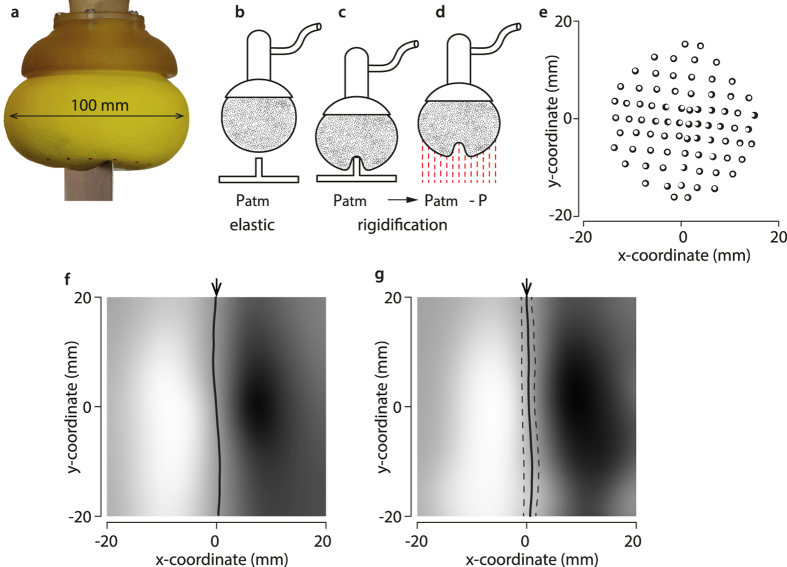
The universal jamming gripper as a tactile sensor. (**a**) Illustrated. (**b**) The gripper membrane was initially in its rest configuration and faced the stimulus straight edge. (**c**) The edge indented the gripper in its elastic state at a given depth. (**d**) Negative pressure rigidified the gripper, the stimulus was removed and its surface scanned in 3D. (**e**) Incremental indentations made it possible to estimate the shear component of the surface deformation by finite differences (black circles of one frame and white circles of the next frame projected on the *x*-*y* plane). (**f**) Shear strain map in gray scale—white for the most negative value and black for the most positive one—with the locus of zero crossing marked by a black line for a single indentation value. Arrows indicate the position of the true edge. (**g**) Shear map averaged over all tested indentations. The average zero crossing locus is indicated by a solid line and the errors (average ± standard-deviation) by dashed lines.

We took advantage of the high conformability capability of the ‘universal jamming gripper’ to make a sensing probe^29^. This device is comprised of an elastic bag filled with a granular material, Fig. 4a. The bag was attached to a rigid base communicating to an air conduit connected to a pressure source, see Fig. 4b. When the pressure inside the bag was lowered, the particles jammed and the system underwent a phase transition from an elastic state to a solid state. The device in its elastic state was pressed against a target object, a close approximation to a line load. When the device conformed tightly with the target, Fig. 4c, pressure was lowered and an imprint of the object was memorized, Fig. 4d. The surface of the probe had 80 optical markers that could be imaged in three dimensions. The shear strain map and the locus of zero-crossing could then be computed by finite differences from successive incremental indentations, as exemplified in Fig. 4e, (*Methods*). The shear strain map would typically have the form shown in Fig. 4f, where the locus of the zero-crossing is shown by a black line (*Methods*).

To test invariance with respect to pressure, the process was repeated for different depths of indentation, Fig. 4g. Over all indentations, the quality of a straight-line regression (Deming method) applied to the zero-crossing points was 0.2 ± 0.1 mm (average ± standard-deviation). The distance of each zero-crossing point to the actual edge was 1.1 ± 1.1 mm and the angular difference between the regressed line and the actual edge was 1.6 ± 1.0 degrees. The locus and the orientation of the edge were recovered regardless of indentation.

## Discussion

Access to the topography of a touched object should be immune to sensing conditions and sensor noise. It should be robust but also efficient. In quasi-static touch, the sources of variability include the contact conditions, the pressure applied, and the sharpness of protruding features. The commonly adopted method of detection of regions of maximal strain by differentiation is inherently unstable because it is sensitive to contact conditions, pressure applied, and sensor noise. The measurement of shear strain rather than normal strain naturally creates a Laplacian of Gaussian-type filter (“Mexican hat” filter) on the spatial distribution of the surface tangential and normal displacements. In other words, shear sensing in an elastic probe provides a scale-space analysis where the detection of zero-crossing makes it possible to reconstruct the topography of a touched object robustly and at different scales.

### Implications for conventional artificial tactile sensing

A tactile sensing principle based on shear strain detection could be applied to artificial tactile sensing with the benefit of invariance to contact conditions, which previous techniques did not afford. Following the approach of previous designs, arrays of shear-sensitive micro-sensors could be embedded within an elastic membrane to detect mechanical stimuli by any known sensing principle (e.g. electrical, optical, or fluidic). Our theory suggests that these individual sensing units ought to be sensitive to shear deformation and relatively insensitive to normal strain.

Tactile sensors often leverage the piezoresistive effect by using arrays of thin conductive elastomer wires or beads that exhibit a variation of conductance under compression^16^,^17^,^18^,^19^,^20^,^21^,^22^,^23^. Such reliance on normal strain renders these devices overly dependent on how an object is pressed upon their surface which introduces noise and ambiguity (see Fig. 3a–d). By contrast, the detection of the zero-crossing of the shear strain would remain invariant under a wide range of sensing conditions. One such important and common case arises when the sensor surface does not conform with an object. Under this scenario, the zero-crossing of the shearing strain would not be different from the case of perfect conformation (see Fig. 3c,d).

### Beyond normal strain

Tactile sensing based on the measurement of parameters beyond just normal strain were considered by Fearing and Hollerbach: “Sensors that combine surface shear sensors, depth strain sensors, and surface deflection sensing would simplify the problem considerably”^40^; see also the discussion by Ricker and Ellis^32^. Tactile sensors that directly detect the displacements of the sensing membrane by optical projection benefit from having direct access to shear strain^41^,^42^. Optics also provides access to shear strain in the bulk, by imaging the relative displacement of two layers^43^. Photogrammic analysis of the light reflected by a surface of constant albedo, can provide detailed topography of a contacted object, and thus shear near the surface^44^. However, to our knowledge, no study has considered the option of detecting the zero-crossing of shear strain, nor explicitly formulated the natural processing performed by mechanical blurring.

### Tactile illusions

In several reported haptic illusions, a sensation of shape can be elicited from distributed cutaneous lateral deformation^45^. For instance, in the “comb illusion”, the sensation of a raised dot traveling on the finger is experienced when a progressive wave of shearing deformation is produced on the fingerpad^33^,^46^,^47^. In the “fishbone illusion” and its variants, a sensation of a raised shape is induced when rubbing one’s finger on surfaces divided into strips made of different materials and/or textures^48^,^49^,^50^. The undulations of a surface can be magnified when a brush-like structure, the “tactile contact lens”, is interposed between the surface and the skin^51^. The recently described “chop-stick illusion” could be similarly explained^52^. All these illusions are based on the principle that certain surface tractions and pressure distributions can produce similar strains. If the surface pressure and traction are such that 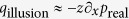 and *p*_illusion_ = cst, where *p*_real_ is the pressure applied by a fingertip pressed against a raised surface, the shearing strain beneath the surface, *γ*_illusion_(*x*, *z*), will be indistinguishable from the shear strain observed in normal conditions, *γ*_real_(*x*, *z*).

### Neurophysiology of edge detection

During static touch, the geometric features of haptic shapes are thought to be encoded by a population of slowly adapting mechanoreceptors^53^. Yet, the computational mechanism accounting for the detection of such features remains obscure. Recently, Pruszynski and Johansson proposed that these computations could be mediated by the combined activity of tactile afferents in the glabrous skin^54^. The authors propose a model based on the convolution of an afferent receptive field map with a spatially-filtered version of the presented stimuli. Under the assumption that slowly adapting mechanoreceptors encode shear strain, our framework suggests that second-order somatosensory neurons could detect the zero-crossing of the sensory input^55^. Thus, geometric haptic feature detection could be performed by a combination of pre-neural and early neural processing. A similar computational mechanism was proposed in vision^9^,^10^,^11^,^37^,^56^, with Laplacian of Gaussian filtering assumed to begin in the neural interaction of ganglion cells of the retina. By contrast, if mechanoreceptors indeed encode shear strain, this filtering process in touch may be directly mediated by the skin deformation. Here, we propose that a simple way for biologic and artificial tactile devices to realize robust edge detection is through shear strain sensing.

## Methods

### Experimental details

It was assumed that the curvature of the probe was much smaller than that of the object, thus approximating the case of a semi-infinite half-space indented by a line load. It was further assumed that the contribution of the tangential displacement to shear was negligible and that the contribution from normal displacement varied little with depth (for validity of these assuptions, see Supplementary Information). Under these approximations, the measurement of the normal displacement gradient at the surface provides an estimate of the shear in the medium close to the surface. We ensured that the edge passed by the center of the probe. We tested nine levels of indentation, the relative distance by which the edge moved after the initial contact with the probe. Indentation was varied by increments of +2.5 mm within a range of 20 mm. The measurements were repeated three times for each level of indentation. From the 3D scans, we computed the shear strain map and its zero-crossing by tracking the markers from two frames incrementally distant from each other. The incremental components, (*u*, *v*, *w*), were then computed over the whole scan area using thin-plate spline interpolation. The shear strain was then estimated by differentiating the interpolated profile.

### Mechanical blurring

The shear stresses caused by concentrated pressure *P* = 1 and concentrated traction *Q* = 1 constant along *y* and applied at *x* = 0 on a linear elastic half space, with *z* pointing inside it, are^38^,


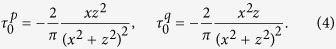


A contact with distributed pressure *p*(*x*) and traction *q*(*x*) can be viewed as an infinite sum of concentrated contacts^38^,


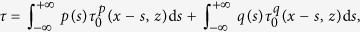


which can be rewritten using the convolution symbol,


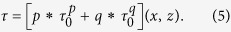


Since we are interested in the character of shearing stress near the contact surface, we can expand the components of shear stress as ‘moment asymptotic expansion’ in the limit 1/*z* → ∞ up to the first order^57^,^58^,^59^. Posing 
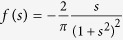
,


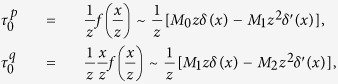


where


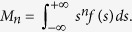


Since the integrands of *M*_0_ and *M*_2_ are odd functions, these moments are equal to zero. Thus, in the limit when *z* → 0,


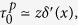



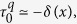


since *M*_1_ = −1. Note that 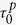 is the function 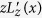 and 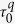 the function 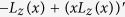, where *L*_*z*_ is the Lorentzian function of scale parameter *z* that converges to the delta function as *z* → 0. To make the blurring nature of contact mechanics explicit, take the case of Gaussian pressure and traction of the form 
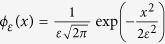
, where *ε* is the width of the Gaussians,


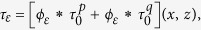


thus,


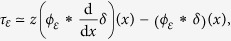


and by the convolution sifting property,


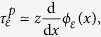



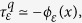


since 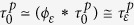 and 
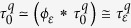
 when *ε* → 0, then from (5), we can express shear stress in terms of Gaussian functions,


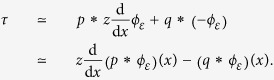


From Hooke’s law applied to an incompressible medium, when *z* is small the shear strain is,


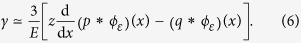


### Blur Size

To evaluate the size of the spread function in the case of a concentrated load as a function of the depth, *z*, we compare the Taylor series expansions of the exact expression of shear stress in the limit when *x* ≪ *z*. From (4),


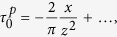


with its blurring counterpart (6),


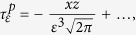


which gives by identification, 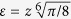. Similarly, the scale of the Gaussian blurring due to a sharp corner can be found by the Taylor expansion of the shear stress expression of a triangularly distributed pressure, 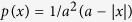, for −*a* ≤ *x* ≤ *a* and *p*(*x*) = 0 elsewhere,


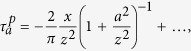


which indicates that blur is both a function of *z* and *a*: 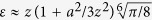, when *a* ≪ *z*.

### Pressure-traction equivalence

From (6), there is a relationship between pressure and traction,


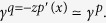


### Displacements

We can express the contact mechanics in terms of displacements since, during interaction with solids, tactile stimuli are specified this way. From elasticity theory^38^, with incompressible media of elastic modulus, *E*, surface pressure and tractions are related to tangential, *u*, and normal, *w*, surface displacements by,


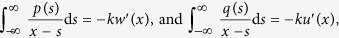


with *k* = 2*πE*/3. Introducing the reciprocal function, *r*(*s*) = 1/*s*, these equations can be rewritten as,


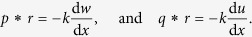


The convolution inverse of the reciprocal function being 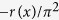, we can write,


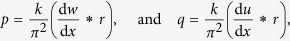


From (6), we have as *z* → 0,


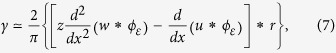


from the differentiation property of convolution. We note that for a uniform tangential displacement, *u*′(*x*) = 0, the zero-crossing of the shear stress, 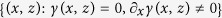, is given at a given depth, *z*, by the solution of


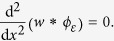


This condition resembles the computation performs in classic visual edge detection techniques, where an edge is detected by computing the zero-crossing of the Laplacian of Gaussian of an image intensity^37^.

## Additional Information

**How to cite this article**: Platkiewicz, J. *et al*. Haptic Edge Detection Through Shear. *Sci. Rep*. **6**, 23551; doi: 10.1038/srep23551 (2016).

## Supplementary Material

Supplementary Information
