# Supplementary material for: Haptic Edge Detection Through Shear
Source: Sci Rep. 2016 Mar 24;6:23551. doi: 10.1038/srep23551 (PMC4806378; doi:10.1038/srep23551)
Supplement: Supplementary Information [file srep23551-s1.pdf]

Supplementary Information

Haptic Edge Detection Through Shear

Jonathan Platkiewicz, Hod Lipson, and Vincent Hayward

Shear Strain From Surface Displacements

Shear strain is related to the components of displacement, u_x and u_z, of any point inside a medium by

$$\gamma = \partial_z u_x + \partial_x u_z.$$

For a concentrated normal linear force density, $P = 1$, along y, the displacements are [1],

$$u_x = \frac{3}{2\pi E}\frac{xz}{x^2+z^2} \quad \text{and} \quad u_z = -\frac{3}{2\pi E}\left[\frac{1}{2}\ln(x^2+z^2) - \frac{x^2}{x^2+z^2}\right] + C,$$

where the constant C depends on the boundary condition. Assuming an incompressible medium, the Poisson ratio was taken to be 0.5. The components of shear are then,

$$\partial_z u_x = \frac{3}{2\pi E}\frac{x(x^2-z^2)}{(x^2+z^2)^2} \quad \text{and} \quad \partial_x u_z = -\frac{3}{2\pi E}\frac{x(x^2+3z^2)}{(x^2+z^2)^2}.$$

Thus, from the previous expressions, we have

$$\partial_z u_x = -\frac{x^2-z^2}{x^2+3z^2}\,\partial_x u_z, \quad \text{so} \quad \partial_z u_x \approx \frac{1}{3}\,\partial_x u_z \quad \text{when} \quad x \ll z. \tag{1}$$

This relationship shows that the contribution of the tangential displacement to the shear strain near a concentrated contact is small compared to the effect of normal displacement. The values in the region around the contact are plotted in Fig. 1.

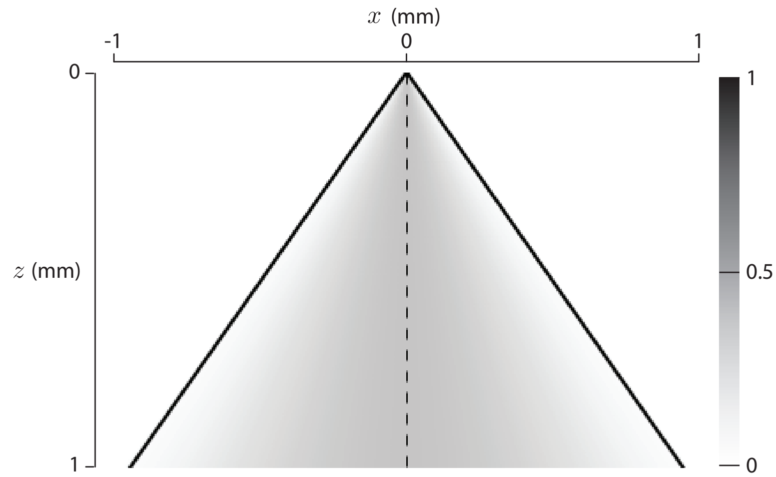

Figure 1: Map of the values of the ratio $\partial_z u_x/\partial_x u_z$. The dashed line represents the line load. The black lines delimit the area where the shear strain is at least 1/10th of its maximal value. Example computed for $E = 7.4$ N.mm^{-2} and for a linear force density $P = 1$ N.mm^{-1}.

References

[1] Johnson KL (1985) *Contact mechanics.* (Cambridge University Press).
